# A Randomised Control Trial of the Impact of a Computer-Based Activity Programme upon the Fitness of Children with Autism

**DOI:** 10.1155/2014/419653

**Published:** 2014-10-07

**Authors:** Kathleen Dickinson, Maurice Place

**Affiliations:** School of Health and Life Sciences, Northumbria University, Coach Lane Campus, Newcastle Upon Tyne NE7 7XA, UK

## Abstract

The poor levels of fitness in children with autism are prompting concern for the children's future health. This study looked to assess if a computer-based activity programme could improve fitness levels (as reflected in cardiopulmonary function) of these children, and achieve a reduction in their body mass index. In a randomised controlled trial, 50 children with autism (of which 33 were under the age of 11 years and 39 were boys) were allocated to an intervention group which encouraged them to use the Nintendo Wii and the software package “Mario and Sonics at the Olympics” in addition to their routine physical education classes. 50 children with autism (34 under the age of 11 years and 40 being boys) acted as controls. At the end of one year, analysis of the changes in scores using analysis of covariance (ANCOVA) on the Eurofit fitness tests showed that the intervention group had made statistically significant improvement on all tests other than flexibility. These improvements were also significantly better than controls. This type of intervention appears to be an effective addition to standard fitness training in order to help children with autism improve their fitness levels.

## 1. Introduction

There is increasing concern about the decline in fitness among children generally and how this reduction increases the risk of ongoing health issues in these children. This fitness decline is associated with a dramatic rise of obesity among the young generally, and since many children and young people with autism have a relatively inactive lifestyle, it has been suggested that this decreased physical activity, together with other factors such as unusual dietary patterns, is the major factor in the relatively greater increased rate of obesity found in children with autism when compared to their typically developing peers [1–5].

Research has shown that increasing the physical activity of children with autism improves general fitness, has a positive impact upon cardiorespiratory function, and offers the potential to control weight gain [6]. Furthermore regular strenuous exercise is associated with decreases in other elements of difficult functioning, such as stereotypical behaviours, hyperactivity, aggression, self-injury, and destructiveness [6].

To achieve this change is however not easy, with the difficulty of encouraging exercise in children with special needs being well discussed in the literature [7, 8] and children with autism presenting a particular challenge in this regard. One method of encouraging physical activity which has been the focus of some interest is the use of computer-based activity programmes. In typically developing children such programmes have been shown to improve the general level of fitness of participants, particularly in those children who have a low level of initial fitness [9, 10]. Theoretically this type of intervention could be of advantage to children with autism because computers are often seen by them as nonthreatening, straight forward, and predictable, as well as demanding minimal social interaction.

In order to evaluate the potential value of computer-based activity programmes to children with autism, a study was undertaken to compare one such programme to standard school-based physical education in the children attending schools with classes specifically for children with autism in the North East of England.

## 2. Methods

The aim of the project was to assess whether additional physical activity in the form a computer-based activity game could improve the cardiopulmonary fitness of children with autism and have a positive impact upon their body mass index (BMI) which also is used as a proxy for fitness. The project was a parallel design with stratification to control for age and gender, and then block randomisation to the intervention and nonintervention groups.

### 2.1. Outcome Measures

One element of the assessment chosen was the multistage progressive shuttle run test (known as the bleep test), which has been shown to be a good measure of cardiopulmonary function because VO_2 max⁡_ scores, a measure of the maximum capacity of an individual's body to transport and use oxygen during incremental exercise, can be calculated from it [11]. Power calculations indicated that a sample size of 34 in each group would allow the detection of a significant difference in VO_2 max⁡_ with a power of 0.9 at a Type I error level of 0.05 for a two-tailed test and an effect size of 0.50 as measured by Cohen's *d*. It was decided to recruit 50 subjects for each group to allow for participant attrition. The secondary outcome assessment was the change in the children's BMI over the duration of the study.

### 2.2. Settings and Participants

Having obtained ethical approval from academic bodies, educational gatekeepers, and parents, the study was carried out over the period of one academic school year (September to June 2011-2012). Three schools in the locale that had classes specific for the teaching of children with autism agreed to take part in the study. The children in these settings ranged in age from 5 to 15 years of age and none had any physical disorder or illness that would reduce their ability to participate in the fitness programme. The families of all the children were approached to participate in the study. After the details of the study had been explained all agreed. The Statements of Special Educational Need for all of the children indicated that they all had an IQ in the moderate/severe range of ability. The children in this pooled sample were then randomly allocated to intervention or nonintervention groups, controlling for age and gender. Selection was completed when 50 families had been allocated to each group. Background information was collected from the family concerning make-up, medical conditions, employment of parents, and so forth.

### 2.3. Fitness Assessment

The physical fitness of the children was evaluated using elements of the Eurofit physical fitness test battery [12], which is a set of nine physical fitness tests covering flexibility, speed, endurance, and strength. These have been shown to have good reliability in typically developing children [13] and have adequate reliability in children with intellectual disability [14]. The five elements of the test that were chosen for this study are described in detail in Table 1. These items were chosen as the smallest number of the 9 tests that would assess the widest range of fitness aspects, while requiring little equipment and being easy to replicate. In fact this group of tests has become well established as the optimum programme for assessment [15].

As described in Table 1, the multistage progressive shuttle run test (known as the bleep test) has been shown to be a good measure of cardiopulmonary function and has been accepted as the standard measure by the British National Coaching Foundation [16]. This test also has the advantage of published norms for typically developing children and offers the opportunity to calculate the VO_2 max⁡_ scores. This is seen as a reliable reflection of physical fitness and uses age as an influencing parameter [11].

### 2.4. Procedure

The testing was carried out in the schools' usual PE areas so the children would be familiar with the spaces. All the schools carried out the tests using the same set procedure, as described in the protocols which accompany each of the tests, and were fully explained to the participating staff. To help the children understand the test elements visual modelling was used together with simplified instructions. To ensure that the children gave their best effort they were instructed to go on until they could go on no longer. In addition, since the teaching staff leading the testing knew the children well they were allowed to explain this requirement in a way they felt the children would best understand.

To overcome the learning effect associated with field-based fitness tests, the children had two trial assessments, and then their scores were recorded at a further assessment, with one of the team (KD) in attendance. The study took place over three academic terms, with the same fitness level tests being repeated at the conclusion of the study period, again under supervision.

In addition to their standard school physical education programme, the children in the intervention group used a Nintendo Wii and the software package “Mario and Sonics at the Olympics.” The participants and staff received an induction in relation to the Wii and associated software games package at the beginning of the intervention. The package was chosen because it had various levels of physical interaction, and each pupil played the game in groups of 2 to 4, under supervision, for a period of 15 minutes per day, three times per week.* Mario and Sonic at the Olympic Games* features authentic Olympic events for the single match and circuit modes. The events closely follow rules and regulations of the specific sports. The types of Wii events are classified as athletics, gymnastics, shooting, archery, rowing, aquatics, fencing, and table tennis. The children taking part in the intervention used the single match mode for up to 4 players. They took part in the athletics, aquatics, fencing, and table tennis. The most popular activities were the running events in the athletics and the swimming events in the aquatics activities. These allowed for four-player use at once. Table tennis allowed for two-player use and the other events were one-player use followed by the next player taking their turn as appropriate. The teachers had flexibility over the time of day the intervention would occur. Wherever possible, the school carried out their intervention at the same time each day so as not to significantly disrupt the children's routine. Random visits were made to the participating schools to monitor the intervention delivery.

Both groups received the standard physical education programme which, in England, is statutorily defined in the National Curriculum for Physical Education [17]. This consists of two PE lessons per week, of 30 or 45 minute duration. In accordance with the requirements of this curriculum these sessions cover sporting activities such as basketball, athletics, gymnastics, and swimming, as well as dance and other ball games, such as cricket.

### 2.5. Additional Assessment

To gain some measure of the dynamics of the families in each of the study groups, the general family functioning of the two groups was compared by asking the children's main care giver to complete the Family Adaptation and Cohesion Evaluation Scales (FACES IV) [18]. This is a self-report measure which gives two scales, one measuring family cohesion (the degree of emotional attachment between family members) and the other flexibility (the ability to change structure, roles, and relationships in response to situational and developmental stress). The scales have proven reliability [19], with moderate levels on each scale being assumed to be more functional than the extreme levels [20].

### 2.6. Sample Make-Up

As can be seen in Table 2, the purposive selection by age and gender resulted in there being 33/34 children under the age of eleven years in the intervention/control groups, and the groups contained 39/40 boys, respectively. All the children had moderate to severe intellectual difficulties, and 35/40 were living with both parents. There were some differences when identifying those that were the only children in the family (9/25), and in terms of parental employment 7/9 came from families where both parents were working, and 13/12 came from families where both parents were unemployed.

All statistical analyses were conducted with IBM-SPSS version 21.0. Normalcy of the fitness and family data was assessed using the Shapiro-Wilk test [21], with the choice of statistical analysis being determined by the results and consideration of the Q-Q plots [22]. Student's *t*-tests were used for comparisons of parametric data and the impact of the intervention being assessed by analysis of covariance (ANCOVA).

## 3. Results

Testing of the family assessment data by the Shapiro-Wilk test confirmed it to be parametric, and the results from the FACES IV questionnaire showed no significant difference in family functioning between the two groups, with the control group mean for cohesion being 57.82 (std dev 12.77) and for the intervention group 60.54 (std dev 11.65,  *t* = 1.11, 98 df). For flexibility of family functioning the mean score for the control group families was 51.3 (std dev 11.71) and the intervention group 50.16 (std dev 10.03, *t* = 0.52, 98 df).

### 3.1. Differences in Initial Parameters

The fitness results showed a wide variation, and the Shapiro-Wilk test confirmed that the results could not be considered to be normally distributed. As can be seen in Table 3, the initial physical fitness levels between the two groups showed some significant differences, with the intervention group being better at the broad jump, bleep test, and sit-up activities and the control group having better scores on the shuttle run test and flexibility.

### 3.2. Changes over Duration of Study

After the study period the children in the control group had shown minor improvements in flexibility, as assessed using the sit and reach test, over the time of the study, but the sit-up score showed little change, as did the broad jump score and bleep test. The control group actually showed a statistically significant increase in their body mass index (BMI), with only four children showing improvement. Also the group's shuttle run times showed an increase (deterioration) with only eight of the fifty improving their scores.

By contrast, the intervention group showed highly significant improvement in all of the fitness measures with, for instance, 46 showing improvement in their shuttle run times, (Table 3). The result that reflects cardiorespiratory fitness and the bleep test improved significantly, and the broad jump and the sit-up scores also showed significant increases. The flexibility median score had undergone a small increase after the intervention (reflecting a deterioration in function), but inspection of the individual scores showed that the majority showed a reduction, with four children whose poorer level of flexibility accounted for the change. The average body mass index (BMI) also showed a reduction in the intervention group, with 39 having a reduced (i.e., improved) score.

### 3.3. Differences between Control and Intervention Groups

When the degree of change seen in control and intervention groups over the time of the study is compared (Table 4), the children in the intervention group showed a significantly more positive level of change in all scores, except flexibility, when compared to the control group (Table 4). 43 had improved their bleep test scores, compared to 13 in the control group, and inspection of the results showed that the change in the control group's shuttle run scores achieved significance because eight children had improved their scores from a high baseline, while 46 of the children in the intervention group had shown improvement in their scores, but to a more modest degree. The broad jump and sit-up scores only improved in the intervention group, while the flexibility score only improved in the control group. With regard to the BMI, a reduction in BMI was taken to be a positive outcome.

To offer some insight as to whether out-of-school activity might bias the fitness results, the parents were asked about the index child's other activities. Only 10 children had any out-of-school activities, four in the intervention group and 6 in the controls.

## 4. Discussion

The potential benefits from using computer-based activity games has been demonstrated in adults [23], but to our knowledge this is the first report of its potential benefit for children with autism. In this study the intervention group showed highly significant improvement in certain aspects of physical activity, particularly in respect to cardiopulmonary fitness (as measured by the VO_2 max⁡_ calculated from the bleep test score). Of perhaps equal note is that the anecdotal feedback from the school staff involved was that all of the children in the intervention group appeared to enjoy participation. One of the measures, the sit and reach activity, which was used to measure flexibility, was not an activity utilised in the computer programme chosen, though in various guises it is part of routine physical education activities. Although there has been some work to show that flexibility improves as general fitness improves [24], significant improvement in flexibility is generally only achieved when flexibility is specifically targeted.

The importance of improving physical fitness is well recognised given its strong association with physical health. Physically active children and adolescents have lower blood pressure levels, more favourable lipoprotein levels, higher bone density, and decreased adiposity compared to their sedentary counterparts [25]. Psychological health and general mental well-being are also improved by such a physically active lifestyle [26–28]. In addition, it has been shown that an active lifestyle has been shown to have a beneficial effect upon psychological health [29, 30], as well as being linked with higher academic achievement [31], especially if the activity improves aerobic capacity [32].

However in recent years there has been a growing concern about the reduction in physical exercise amongst children [33] and that this is increasing the risk of significant health problems for them in later life particularly if cardiorespiratory fitness is poor [34]. This is in part because active and energetic children tend to remain active throughout middle and late adulthood [35], whereas sedentary children are more likely to become sedentary adults [36].

Children with autism have greater difficulties than typically developing children with participating in physical activity because of the nature of their difficulties. Although the literature on physical activity levels in children with autism is sparse [37], several studies have found that children with autism are less active than children without disabilities [38, 39], and even when children with autism achieve recommended activity levels during physical activity lessons, they are significantly less active during break times [40–42] and when at home [37]. Also, when compared to young people without autism, young people with autism are more likely to have difficulties with balance, postural stability, gait, joint flexibility, and movement speed [43], all of which adds to their difficulty in participating in physical activity.

As with typically developing children, regular physical activity has been shown to reduce the BMI of children with autism [44] and reduce stereotypical and self-stimulating behaviour [45]. Physical activity has also been found to help improve other behavioural issues [24, 44, 46]. The impact however can be even wider, with improvements in social behaviour [47] and academic engagement also being reported [48]. Finally, at least one study has shown that physical exercise reduced stress levels, as measured by salivary cortisol and self-report measures, in children with autism; however, these improvements quickly faded when the exercise programme ceased [49].

These findings strengthen the conclusion that it is important for these children to have a continuous programme of exercise, with a method of delivery that maintains the child's engagement. There is evidence that children with autism have difficulty understanding the goals of physical exercise [50], increasing further the challenge of ongoing involvement with fitness programmes. The research that does exist on helping children with autism take more exercise and improve their physical fitness tends to be focussed upon jogging and swimming [45, 51], with modelling and physical guidance generally being the method of instruction [45]. However to achieve regular participation there must be some source of external regulation [52], and the activities of a nature that will engage them sufficiently such that they will wish to participate.

While there are an increasing number of computer-based programmes that offer guided fitness activities, programmes that are game-based have been viewed as being of little value to health and well-being. Indeed video games have often been blamed as contributing factors to the increasing obesity levels [53]; however, it is being increasingly shown that physical activity-focussed programmes increase the activity levels of participants [54]. This recognition has led to their use in various settings, with activity-based computer programmes making a positive impact upon physical rehabilitation after an accident [55], as well as reducing mild cognitive impairment in older adults [56]. There is also a growing evidence base in adults that computer- and web-based interventions are effective at promoting physical health more generally [23]. That such programmes would also be helpful in children would not be surprising [57] and perhaps particularly so in children with autism because computer-based interventions offer a visual and nonjudgemental opportunity for them to take part in games that make no social interaction demands.

In any such study there are limitations to the conclusions that can be drawn. In this case the sample was drawn from a small geographical area in the North of England which is urban in nature, making it difficult to be confident about how much the findings would represent the impact upon children with autism in other areas. Also, although efforts were made to match the two groups, the relatively small population from which to draw meant that the matching process could only use a limited number of parameters. By choosing age and gender the possibility of differences in initial fitness levels between the groups was accepted, and in the event this proved to be the case. However it was anticipated that the potential impact of this upon the results could be reduced by using nonparametric statistical analyses and the degree of change in scores as the main outcome measure. Using a pooled sample from three schools gave rise to a potential contamination between control and intervention children, but it was decided to proceed with this method because it gave a degree of control about wider school issues and reduced the extra sessions that the staff in each school had to undertake. The staff had been asked not to alter the standard PE lessons that all the children provided, but the project resources did not permit the content of these to be monitored. It was recognised that the ongoing research may have prompted some change in content of the routine PE sessions, but it was hoped that the fact that both control and intervention children were sharing these lessons would minimise the impact.

In addition, it is known that a child's level of physical activity is strongly influenced by family issues such as parental expectations and support, the modelling of siblings, and the opportunities that exist within their community to participate in physical exercise [58, 59]. In this study resources did not permit these specific issues to be explored in any detail, but the small number of children with out-of-school activities and the larger number being in the control group suggested this was unlikely to exert a negative effect upon the overall results.

## 5. Conclusion

The growing concern about deteriorating fitness levels and their impact upon children's health has a special significance for children with autism because of the way their patterns of functioning makes it difficult to encourage their participation in standard school physical education activities and how prone they are to become obese, further reducing their capacity to exercise. The results from this study indicate that an improvement in specific fitness measurements and BMI can be achieved by children with autism if they follow a computer-based activity programme (in this particular study the “Mario and Sonic at the Olympics” for the Nintendo Wii). Such an intervention is a practical option for schools, but the finding that some aspects of fitness did not improve emphasises the importance of offering such computer-based activities as part of a wider physical fitness regime so that physical functions that such programmes do not address can be tackled through other activities.

## Figures and Tables

**Table 1:** Fitness tests used in the study.

Test	Area of measurement/explanation	Equipment required
Body mass index	Body fat measurement	Height scale and weighing scales

National Coaching Foundation (NCF), multistage fitness test-progressive shuttle run test known as the bleep or beep test.	Cardiorespiratory fitness (aerobic) VO_2 max⁡_. This is a maximal test. The bleep test is a maximal exercise test to measure aerobic fitness. It involves running up and down a 20 m track in time to increasingly faster beep signals.	Test CD and CD player or MP3 download, 20 m running space, marker cones.

Standing long jump test (broad jump)	Measurement of explosive leg strength. A starting line is marked on the floor and the participants have to take a standing two-footed jump from this line with the aim to jump as far as they can. The arms may be used to aid the jump.	2 m of space. Marker for starting line. Tape measure. Or Standing broad jump kit.

10 × 5 m shuttle test	Measurement of speed and agility and aerobic capacity. Subjects run between two markers of 5 m; there and back counts as 1 repeat and this is continued until 10 repeats have been carried out.	Cones, tape measure to mark out 5 m, stop watch.

Partial curl up test known as sit-ups	Measurement of abdominal strength and endurance. The child lies on the floor with knees flexed. The subject curls up his/her trunk then lowers back to the floor, repeating this as many times as they can. The test lasts for duration of 30 seconds.	Mat for the child to lie on. Stop watch. Additional person to hold legs or time test.

Sit and reach test	Test measures the flexibility of the lower back and hamstring muscles, that is, hip flexibility. The test involves sitting on the floor with legs out straight ahead and then reaching as far forward as possible.	Sit and reach board/equipment or tape measure.

**Table 2:** The demographic information of the sample.

	Intervention group (*n* = 50)	Control group (*n* = 50)
Boys		
≤10 yrs	27	27
≥11 yrs	12	13
Girls		
≤10 yrs	6	7
≥11 yrs	5	3
Only child	9	25
Living with both parents	35	40
Both parents working	7	9
Both parents unemployed	13	12

**Table 3:** Initial and follow-up fitness results as medians and interquartile range (IQR) scores (*Z* calculated using Wilcoxon signed rank test between time samples and Mann Whitney *U* for difference between initial scores of control and intervention groups).

	Control group			Intervention group			Difference between initial scores of control and intervention groups (*Z*)
	Median (IRQ)		Difference between initial and follow-up scores (*Z*)	Median (IRQ)		Difference between initial and follow-up scores (*Z*)
	Initial score	Follow-up score		Initial score	Follow-up score	
Body mass index (BMI)	20.2 (5.6)	21.5 (5.5)	4.86∗∗∗	20.1 (6.1)	19.8 (5.6)	−5.37∗∗∗	0.72
Bleep test	2 (3)	2 (2)	1.61	3 (4)	4.5 (5)	5.92∗∗∗	2.52∗
Shuttle run	84.5 (49)	90 (42)	4.04∗∗∗	92 (27)	68 (43)	−4.89∗∗∗	2.33∗
Broad jump	54.5 (23)	52.5 (20)	−0.77	80 (41)	92.5 (50)	5.12∗∗∗	4.59∗∗∗
Sit-ups	7.5 (5)	7.5 (4)	1.94	10 (10)	13 (7)	5.08∗∗∗	2.27∗
Flexibility	10 (6)	10 (8)	2.48∗	8 (13)	9 (13)	0.09	2.05∗

*P < 0.05, **P < 0.01, and ***P < 0.001.

**Table 4:** Comparison of changes to fitness scores between the control and intervention groups (*F* calculated using ANCOVA with fixed factors of age and gender).

	Number of children showing improvement in score		Significance in difference of degree of change between control and intervention groups (*F*(1,90))
	Control group (*n* = 50)	Intervention group (*n* = 50)	
VO_2 max_	13	43	16.13∗∗∗
Body mass index (BMI)	4	39	30.06∗∗∗
Bleep test	13	43	13.15∗∗∗
Shuttle run	8	46	41.35∗∗∗
Broad jump	18	39	40.30∗∗∗
Sit-ups	9	38	25.72∗∗∗
Flexibility	4	4	1.20

*P < 0.05, **P < 0.01, and ***P < 0.001.
